# Sen-2 LULC: Land use land cover dataset for deep learning approaches

**DOI:** 10.1016/j.dib.2023.109724

**Published:** 2023-10-24

**Authors:** Suraj Sawant, Rahul Dev Garg, Vishal Meshram, Shrayank Mistry

**Affiliations:** a Geomatics Engineering, IIT Roorkee, Uttarakhand 247667, India; b COEP Technological University, Pune, Maharashtra 411005, India; c Vishwakarma Institute of Information Technology, Pune, Maharashtra 411048, India

**Keywords:** Land Use Land Cover (LULC), Remote sensing, Satellite imagery, Sentinel-2, Deep learning, Convolution neural network, Image classification

## Abstract

Land Use Land Cover (LULC) classification is pivotal to sustainable environment and natural resource management. It is critical in planning, monitoring, and management programs at various local and national levels. Monitoring changes in LULC patterns over time is crucial for understanding evolving landscapes. Traditionally, LULC classification has been achieved through satellite data by remote sensing, geographic information system (GIS) techniques, machine learning classifiers, and deep learning models. Semantic segmentation, a technique for assigning land cover classes to individual pixels in an image, is commonly employed for LULC mapping. In recent years, the deep learning revolution, particularly Convolutional Neural Networks (CNNs), has reshaped the field of computer vision and LULC classification. Deep architectures have consistently outperformed traditional methods, offering greater accuracy and efficiency. However, the availability of high-quality datasets has been a limiting factor. Bridging the gap between modern computer vision and remote sensing data analysis can revolutionize our understanding of the environment and drive breakthroughs in urban planning and ecosystem change research. The "Sen-2 LULC Dataset" has been created to facilitate this convergence. This dataset comprises of 213,761 pre-processed 10 m resolution images representing seven LULC classes. These classes encompass water bodies, dense forests, sparse forests, barren land, built-up areas, agricultural land, and fallow land. Importantly, each image may contain multiple coexisting land use and land cover classes, mirroring the real-world complexity of landscapes. The dataset is derived from Sentinel-2 satellite imagery sourced from the Copernicus Open Access Hub (https://scihub.copernicus.eu/) platform. It includes spectral bands B4, B3, and B2, corresponding to red, green, and blue (RGB) channels, and offers a spectral resolution of 10 m. The dataset also provides an equal number of mask images. Structured into six folders, the dataset offers training, testing, and validation sets for images and masks. Researchers across various domains can leverage this resource to advance LULC classification in the context of the Indian region. Additionally, it catalyzes fostering collaboration between remote sensing and computer vision communities, enabling novel insights into environmental dynamics and urban planning challenges.

Specifications Table

**Table:** 

Subject	Computer Science (Artificial Intelligence), Remote Sensing, Geographical Information Systems, Geospatial Analysis
Specific subject area	Image analysis & classification, Semantic Segmentation, useful for landscape ecology and sustainable agriculture
Data format	Raw Analysed Filtered
Type of data	Images
Data collection	Satellite images are acquired from the Sentinel-2 Level-2A product database provided by the Copernicus Open Access Hub (https://scihub.copernicus.eu/) platform. The images in the dataset are extracted from four tiles of the Central Indian region. The tiles are downloaded for the month of February and March 2021.
Data source location	Country: India All the downloaded images are Sentinel-2 L2A products downloaded from the Copernicus Open Access Hub (https://scihub.copernicus.eu/) platform.
Data accessibility	Repository name: Mendeley Data, Data identification number: 10.17632/f4ky6ks248.2 Direct URL to data: https://data.mendeley.com/datasets/f4ky6ks248/2

## 1 Value of the Data

• The dataset of 213,761 pre-processed 10 m resolution images representing seven distinct Land Use Land Cover classes with equal numbers of mask images are generated.
• This dataset encompasses seven critical LULC categories: Water bodies, dense forest, sparse forest, barren land, built-up, agricultural land, and fallow land, which helps drive advancements in LULC classification research.
• This dataset is designed for LULC classification of the Indian region, facilitating the development of deep learning models.
• This dataset could be used to train, validate, and test machine learning models for LULC classification models.
• No comparable dataset of this nature is available in the existing literature for this specific problem. Moreover, even when other datasets are available, the distinctive value of this dataset lies in its specific focus on seven distinct classes, making it a valuable complement to existing datasets and a critical resource for research enthusiasts.

## 2 Data Description

Land Use Land Cover (LULC) classification is critical to remote sensing and environmental monitoring. It entails classifying the Earth's surface into many groups depending on the numerous forms of land cover, such as forests, agriculture, urban areas, water bodies, and more. These details are essential for various applications, including urban planning, agriculture management, natural resource conservation, and disaster assessment.

LULC datasets are valuable resources for LULC classification and plays a pivotal role in assessing the accuracy of Machine Learning (ML) and Deep Learning (DL) classifiers, particularly for remote sensing and geospatial applications. They provide a standardized and comprehensive means of evaluating classifiers, allowing researchers and practitioners to make informed decisions and implement more accurate and reliable LULC classification models for various applications. Table 1 shows the feature comparison of proposed [1] dataset with similar existing datasets and justifies the need for proposed. It is essential to consider the limitations of existing datasets when choosing a dataset for specific research objectives. Additionally, understanding each dataset's geographic and thematic coverage is crucial for selecting the most suitable dataset for a particular application or study area.

**Table 1:** Comparison with other datasets.

Sr. No.	Dataset	Description
1	SeasonNet [2]	This dataset is designed for monitoring seasonal changes in land cover. It focuses on capturing images of the same location across different seasons. As it focuses on seasonal changes in land cover, it may not be suitable for non-seasonal or long-term studies.
2	MultiSenGE [3]	It aims to support the development of models that can generalize across different satellite sensors and is particularly useful for cross-sensor studies. Combining data from multiple sensors can be challenging and requires additional preprocessing.
3	LandoverNet [4]	This dataset focuses on land cover classification using Sentinel-2 satellite data. It contains classes primarily related to agriculture and land use, which covers the partial spectrum of land cover types in more diverse regions.
4	BigEarthNet [5]	It is designed for LULC classification and prediction tasks using Sentinel-2 satellite data, covering large areas and leading to large data volumes. Analyzing and processing the data can be computationally intensive, potentially requiring substantial computing resources.
5	SEN12MSI [6]	This dataset provides data from four different satellite sensors (Sentinel-1, Sentinel-2, Landsat 8, and MODIS) and is designed for LULC classification and change detection. Integrating and harmonizing data from multiple sensors can be complex and may require careful preprocessing.
6	EuroSAT [7]	It is designed for LULC classification using Sentinel-2. It is explicitly designed for LULC classification over European regions, limiting its applicability for research in other parts of the world.
7	Sen-2 LULC (Proposed) [1]	This dataset is designed for the Indian region. It has total seven classes. It has balanced instances of all the classes.

Considering the need of generating the dataset for Indian region, we introduce the “Sen-2 LULC Dataset” [1] to support the interdisciplinary convergence. This dataset will help the researchers to perform the LULC classification using Machine Learning and Deep Learning approaches. Further these LULC maps can be used for the applications like route optimization, change detection, flood detection [8,9], crop zonation [10], detecting oil spill [11], and detecting different objects from hyperspectral images [12]. This extensive dataset comprises 213,761 meticulously pre-processed images at a 10 m resolution, capturing the diversity of seven distinct LULC classes: water bodies, dense forests, sparse forests, barren land, built-up areas, agricultural land, and fallow land. Each image depicts multiple coexisting land use and land cover classes, faithfully reproducing the intricate real-world landscapes. The dataset originates from Sentinel-2 satellite imagery, acquired via the Copernicus Open Access Hub, with image capture spanning the months of February and March 2021. It encompasses spectral bands B4, B3, and B2, corresponding to the RGB channels, providing a spectral resolution of 10 m. The dataset is organized deliberately into three folders as:

1. Training: The subset of data that is utilized to fit the model. This data is seen by and used to train the model. The training dataset comprises 70 % (149,601) of the total images in the collection.
2. Validation: A portion of dataset used to tweak model hyperparameters and give a fair evaluation of how well a model fits the training dataset. This dataset's validation dataset contains 15 % (32,080) of the total images.
3. Test: This dataset is utilized to evaluate the model's ability to match precisely the training dataset. It contains 15 % (32,080) of the images in the original dataset.

**Fig. 1:**
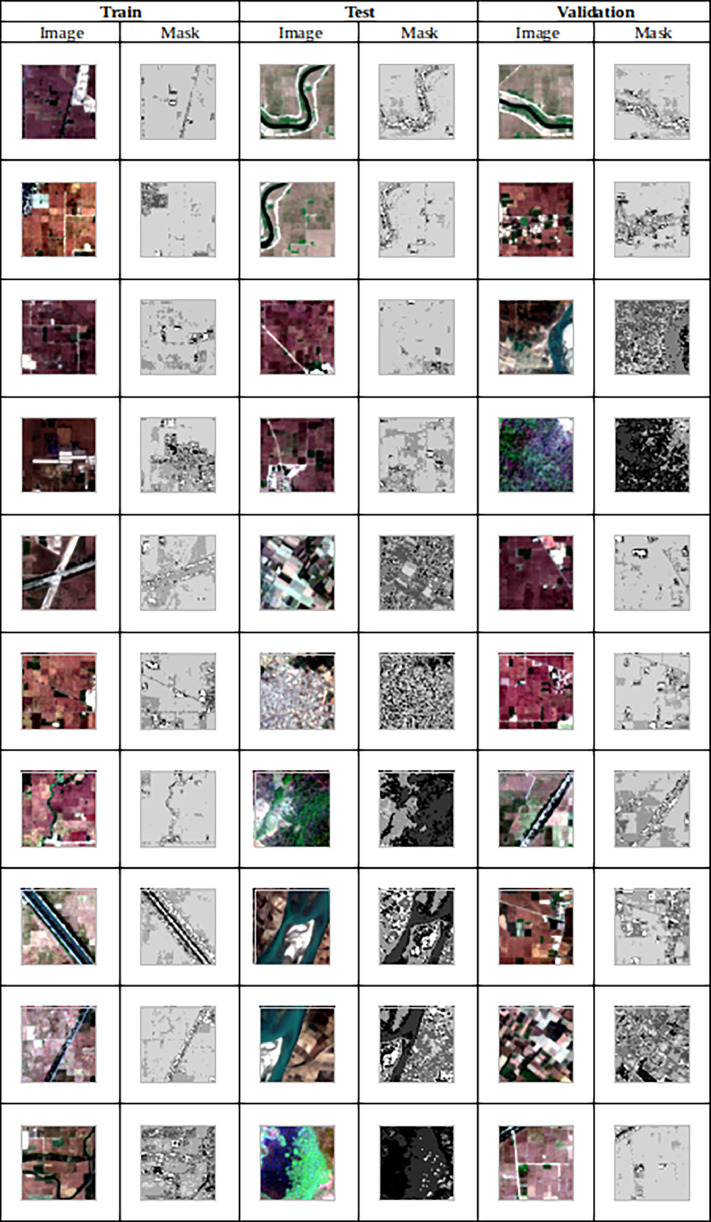
Sentinel-2 image (left). Generated mask (right) in Training, Testing and Validation folders.

Additionally, the dataset includes an equal number of mask images. This dataset offers distinct sets for training, validation, and testing, encompassing images and their corresponding masks. The Fig. 1 shows the sample images in the dataset consisting of images and masks.

## 3 Experimental Design, Materials and Methods

We have taken four tiles from across India to represent the diversity of data. They are chosen such that they include a maximum number of classes mentioned in the work. The images considered in this dataset are Sentinel-2 Level-2A product, multi spectral satellite images with 10 m resolution and cloud cover ranging from 0 to 0.5%. Semi-Automatic Classification Plugin (SCP) available in Quantum GIS (QGIS) is used to create the labeled data. Different region of interest (ROIs) polygons have been identified from the selected tiles. The spectral signatures from these ROIs are extracted to generate the training set. The training file generated is used by SCP, which uses the Spectral-Angle mapping algorithm [13] to classify the LULC classes for the selected tiles. The accuracy assessment is done by visiting the location of images taken. Procedure applied in generation of dataset is summarized in four steps as follows:

### 3.1 Acquiring Sentinel-2 Tiles

Copernicus Open Access Hub [14] provides various products from the Sentinel mission, such as Sentinel-1,2,3, and Sentinel-5P, free and unrestricted access to users for download. Sentinel-2 L2A atmospherically corrected images are downloaded from February and March 2021. Platform, processing level, cloud cover, acquisition dates, and region of interest are some parameters provided to download the required satellite images for our study area. Sentinel-2 tiles are acquired by manually downloading tiles from the Copernicus Hub.

### 3.2 Data Pre-Processing

Sentinel-2 L2A satellite images downloaded are atmospherically corrected and require minimum pre-processing. Each of the acquired tiles consists of 13 bands belonging to different resolutions. The resolution of these bands ranges from 10 m to 60 m; for selecting the optimal three bands from the 13 bands, the Optimum Index Factor (OIF) [15] statistic value is computed. Based on the maximum OIF value, Bands B4, B3 and B2 are finalized. Different pre-processing techniques, such as mosaicking, layer-stacking, and clipping, are performed on the Sentinel-2 bands for the selected tiles. Open-source Geospatial Data Abstraction Library (GDAL), a python library performs various pre-processing tasks, which is used for writing and reading vector and raster formats of geospatial data. After selecting tiles for our study area, mosaicking is done to merge the individual tiles for every single band, followed by layer-stacking, which combines the bands by stacking them one on top of the other to create a single satellite image comprising multiple bands. Finally, clipping is done on the satellite image, which crops the image to generate the required study area.

### 3.3 LULC Classification Using Open Source GIS Tool

QGIS [16,17] provides the Semi-Automatic Classification Plugin (SCP) [18] that provides the interface in QGIS to generate training data for the task of LULC classification. A satellite image is loaded in QGIS, and various Regions of Interest (ROIs) polygons are identified belonging to seven LULC classes: barren land, water bodies, sparse and dense forest, built-up areas, fallow land, and agricultural land. The different ROIs belonging to different LULC classes contain different spectral signatures used to create the training dataset. The training set generated from ROIs using SCP includes the scikit-learn Python library, which uses the Spectral Angle Mapping algorithm to generate LULC classification for the entire satellite image. The ground truthing is done by visiting the location personally. The misclassified pixels were then identified and correctly classified by comparing with the ground truth. The LULC classes denotation is described in Table 2.

**Table 2:** LULC classes denotation.

Class Label	Class denotation	Description
0	Unclassified	Pixel that is not classified is assigned unclassified name and zero label.
1	Water Bodies	Water from streams, rivers, lakes and reserviors.
2	Dense Forest	Area where tree cover canopy density is in between 40 and 70 %.
3	Built up	Artificial / concrete surface
4	Agriculture land	Area where crops are cultivated or planted vegetation.
5	Barren land	Land where crops or plants can not be cultivated due to infertility of the soil.
6	Fallow land	Land under agricultural cultivation but currently kept unclutivated
7	Sparse Forest	Area where tree cover canopy density is in between 10 and 40 %.

### 3.4 Patches Generation

**Fig. 2:**
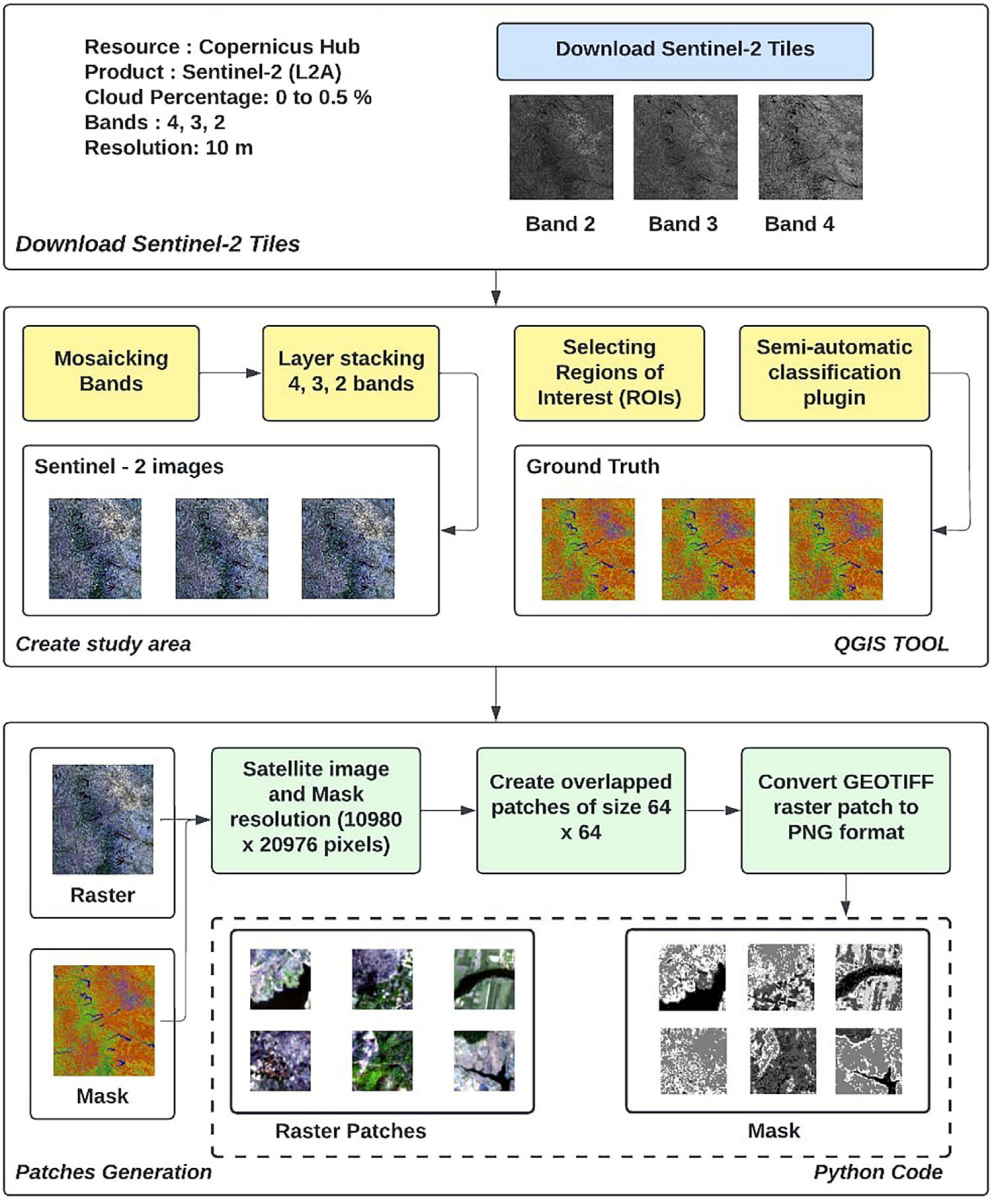
Data set generation steps.

The selected tiles and their corresponding labeled mask are large in dimension and cannot be used to train deep learning architectures; therefore, there is a need to create small patches of the entire dataset. At first, patches of 256 × 256 were created, but the image dataset was too small, and the generated patches were non-overlapping; thus, the models did not perform well. As the dataset generated was not diverse and large enough for training the CNN, we generated patches of size 64×64. Rasterio 19 and GDAL python libraries are used to create patches of size 64×64. Extents or bounds that define the limits of satellite images are known as Geospatial data boundaries. Rasterio python library is used to get the extent of satellite images, which traverse the image horizontally and vertically to generate individual patches of size 64×64 using the GDAL translate function. The function takes the four extents of a small generated patch from existing satellite images and corresponding masks. A total of 213,761 patches are created, of which 70 % patches, are considered for training, and the remaining 30 % are equally divided into validation and testing. Fig. 2 represents the workflow of how the dataset is created step-by-step.

The proposed dataset is evaluated on three Deep Learning models with different backbones. The basic model used is Unet architechture with ResNet50, ResNet101 and ResNet152 as backbones. Table 3, Table 4, Table 5 shows the results obtained. The models are executed for 100 epochs with learning rate of 0.01. Models are executed on Nvidia DGX-1 Workstation with 32GB of Graphics Processing Unit.

**Table 3:** Results of proposed dataset on UNet-ResNet50.

	Water	Dense Forest	Sparse Forest	Barren land	Built up	Agriculture Land	Fallow Land
Precision	0.56	0.61	0.75	0.74	0.85	0.87	0.74
Recall	0.46	0.51	0.63	0.73	0.81	0.96	0.65
Over all Accuraccy	0.98	0.99	0.96	0.94	0.9	0.92	0.97
F1- Score	0.5	0.56	0.69	0.73	0.83	0.91	0.7
MCC	0.5	0.55	0.67	0.7	0.76	0.85	0.68

**Table 4:** Results of proposed dataset on UNet-ResNet101.

	Water	Dense Forest	Sparse Forest	Barren land	Built up	Agriculture Land	Fallow Land
Precision	0.54	0.50	0.73	0.69	0.84	0.87	0.63
Recall	0.52	0.56	0.61	0.70	0.79	0.94	0.56
Over all Accuraccy	0.98	0.99	0.96	0.93	0.89	0.92	0.96
F1- Score	0.53	0.53	0.67	0.70	0.81	0.90	0.59
MCC	0.52	0.52	0.65	0.66	0.73	0.83	0.57

**Table 5:** Results of proposed dataset on UNet-ResNet152.

	Water	Dense Forest	Sparse Forest	Barren land	Built up	Agriculture Land	Fallow Land
Precision	0.51	0.69	0.73	0.70	0.79	0.90	0.77
Recall	0.46	0.57	0.56	0.74	0.81	0.93	0.67
Over all Accuraccy	0.98	0.99	0.95	0.94	0.88	0.92	0.97
F1- Score	0.49	0.62	0.64	0.72	0.80	0.92	0.72
MCC	0.48	0.62	0.62	0.68	0.72	0.84	0.70

The results show that the majority of classes are having acceptable results, ensuring beneficial and reliable dataset. Differentiation between dense and sparse forest is confusing. Due to the extensive complexity, these two classes are still difficult in its interpretation. This can be seen in the results shown above. The challenge is due to similarity in the two classes and very negligible difference in the structure. Additional ground truth information is needed to properly define the class and enhance the discrimination of the class boundaries. In addition, if possible, additional indices may be added to strengthen the structural constraint of the strategy of classification. The modelwise results are shown in Table 6.

**Table 6:** Results of proposed dataset on deep learning models.

	UNet-ResNet50	UNet-ResNet101	UNet-ResNet152
Precision	0.73	0.69	0.73
Recall	0.68	0.67	0.68
Overall Accuraccy	0.95	0.95	0.95
F1- Score	0.70	0.68	0.70
MCC	0.67	0.64	0.67

Considering the seven classes of the LULC, the generated dataset has given the Overall Accuracy of 95 % for all the three deep learning models.

This dataset is generated for LULC Classification using Deep Learning models, so that the obtained LULC map will be used as one of the input layers for Gas Pipeline Route Optimization using Artificial Intelligent techiniques. This dataset may be used to enhance the accuracy of LULC maps generated, so that this map can be used for route optimization route planning, change detection applications.

## 4 Limitations

The dataset contains the images from four different tiles of the Indian region. This may add the limitation of not having the variety of samples from different regions. Every possible care is taken to provide the most accurate mask, but as most of the regions are mountainuous and covered with dense forest, ground truthing can not be done for every region, that may result in loss of accuracy upto minimal extent. This dataset can be used for the applications that uses seven specified classes only.

## Ethics Statement

The current research, according to all of the authors, does not use any data gathered from social media platforms, human subjects, or experiments involving animals.

The images used in the dataset are downloaded from European Space Agencey (ESA) Copernicus Hub. ESA grants permission to use this data for research purpose and non commercial use.

## CRediT authorship contribution statement

**Suraj Sawant:** Conceptualization, Visualization, Methodology, Investigation, Data curation, Writing – original draft, Writing – review & editing. **Rahul Dev Garg:** Supervision, Formal analysis. **Vishal Meshram:** Data curation, Supervision, Validation, Writing – review & editing. **Shrayank Mistry:** Data curation.

## Data Availability

Sen-2 LULC (Original data) (Mendeley Data).
